# Strategies of Bacterial Over Expression of Membrane Transporters Relevant in Human Health: The Successful Case of the Three Members of OCTN Subfamily

**DOI:** 10.1007/s12033-012-9586-8

**Published:** 2012-07-28

**Authors:** Cesare Indiveri, Michele Galluccio, Mariafrancesca Scalise, Lorena Pochini

**Affiliations:** Department of Cell Biology, University of Calabria, Via P.Bucci 4c, 87036 Arcavacata di Rende, Italy

**Keywords:** Over expression, *E. coli*, Membrane proteins, Membrane transport, OCTN, Organic cations, Carnitine, Human health, Crohn’s disease

## Abstract

The OCTN subfamily includes OCTN1, 2, and 3 which are structurally and functionally related. These transporters are involved in maintenance of the carnitine homeostasis, which is essential in mammals for fatty acid β-oxidation, VLDL assembly, post-translational modifications, and other essential functions. Indeed, defects of these transporters lead to severe pathologies. OCTN1 and OCTN2 are expressed in many human tissues, while OCTN3 gene has been identified only in mouse and rat. The transporters mediate transport of carnitine and other substrates with different efficiencies and mechanisms. In order to over express the three proteins, a screening of many combinations of *E. coli* strains with plasmid constructs has been conducted. Only Rosetta(DE3) or Rosettagami2(DE3) gave significant expression. Higher protein amounts were firstly obtained with pET-41a(+) or pGEX-4T1 carrying fusion protein tags which required additional purification passages. Vectors carrying only a 6His tag, suitable for single passage purification, were preferred even though they lead to lower initial expression levels. Expressions were then increased optimizing several critical parameters. hOCTN1 was obtained with pH6EX3 in RosettaGami2(DE3)pLysS. hOCTN2 and mOCTN3 were obtained using pET-21a(+) in Rosetta(DE3). In particular, hOCTN2 was expressed only after codon bias, substituting the second triplet CGG with AAA (R2K mutant). The best growth conditions for hOCTN1 and mOCTN3 were 28 °C and 6 h of induction, while 4 h of induction for hOCTN2R2K. The proteins collected in the insoluble fraction of cell lysates, solubilized with sarkosyl, were purified by Ni-chelating chromatography. Final yield was 2.0, 3.0, or 3.5 mg/l of cell culture for mOCTN3, hOCTN1, or hOCTN2R2K. The data indicated that, in spite of the close evolutionary relations, several factors play different critical roles in bacterial expression of the three proteins, thus general criteria cannot be underlined. However, the strategy of dealing with related proteins revealed to be finally successful for over expressing all the three subfamily members.

## Introduction

Not very long ago (70’s) it was believed that many physiological or xenobiotic compounds cross biological membranes by simple diffusion. This assumption has been now upset. The profound modification of the point of view is a consequence of the exponential increase of membrane transporter studies since that time. Indeed, from 70’s the papers published per year dealing with membrane transport raised by about four fold to nowadays (results on the basis of occurrence of “membrane” and “transport” in title field on http://www.ncbi.nlm.nih.gov/pubmed). It has been thus assessed that membrane transport systems, with a very limited number of exceptions, are necessary for uptake, elimination, and intracellular trafficking of virtually all nutrients and metabolites, and hence transporters are key components for maintaining and regulating cell homeostasis. Thus, many of these proteins are essential for life. Besides the development of methodologies for studying transport systems, other important achievements stimulated the study of membrane transporters especially during the last two decades: (i) Genome wide sequencing revealed that genes coding for membrane proteins are much more abundant than previously thought. As an example, the genome of *H. sapiens* contains more than thousand genes coding for membrane transporters (http://www.membranetransport.org/) which account for about 3 % of the total ORFs. (ii) Defects of transporter functions have been correlated to several human pathologies of a wide range of severity [1–6]. (iii) Recently it emerged that transporters have high relevance in drug delivery, absorption, and side effect onset representing the first contact point with the organism [7–9]. However, many structures of bacterial transporters but no structure of human and very few of mammalian transporters have been resolved so far (http://www.membranetransport.org/). This is due to the lack of methodologies for over expressing and folding membrane transporters. Indeed, the mitochondrial ADP/ATP transporter, which is a single case of mammalian secondary active transporter entirely crystallized so far, was obtained by purification from bovine heart since it is one of the most abundant mitochondrial proteins [10]. On the basis of the above considerations, strategies aimed to solve the problems hampering protein expression and to study structure/function relationships of transport proteins related to human pathology are particularly welcome to both transport protein and human health scientific communities.

## Problems With Functional and Structural Studies

Methodologies to obtain large amount of folded transport proteins are essential for studying their structure and structure/function relationships. Each of the strategies currently used for producing transport proteins obviously presents advantages and disadvantages. The features of each methodology have to be considered as function of the specific protein and of the studies to be carried out. This will help the choice of the appropriate experimental systems. This argument has been previously well reviewed [11]. In principle, eukaryotic expression systems would be better for obtaining correctly folded proteins. However, these systems, with very few exceptions, give lower yields with respect to bacterial ones. Hence, proteins obtained by expression in eukaryotic organisms are normally not sufficient for structural studies. Thus, several efforts are in progress to find suitable conditions for bacterial expression which remains the method of election for recovering high amounts of proteins. This system also allows to perform minimal modification of the proteins, such as His tag insertion that facilitates the further purification step, which is also essential for structural and functional studies.

For its low costs, harmless for humans, fast growth, easy handiness, and wide empirical experience in its usage, *E. coli* is the most used microorganism for protein expression. However, there are still a lot of unsolved problems limiting the expression of mammalian membrane transporters and, in general, of hydrophobic proteins. These problems are mainly caused by differences in both DNA composition and protein structure between bacteria and mammals. *E. coli* strains have been originally engineered to improve mammalian protein expression. Examples are *E. coli* BL21(DE3) and K-12 from which other optimized strains were derived [12, 13].

Two clue points in which it can be possible to act for obtaining increase of heterologous membrane protein expression are listed below:

### Codon Usage and Host Choice

An important challenge for heterologous expression is the different codon usage of mammals and in particular humans, which causes difficulties in expressing proteins in bacteria [14]. For example, *E. coli* does not use certain codons (such as AUA for Ile, or AGA and AGG for Arg) that are frequently used in mammalian genes [15]. This problem can be at least partially overcome by modifying the host; accordingly, *E. coli* strains engineered with tRNA frequently used in humans, such as Rosetta(DE3) and BL21-CodonPlus RIL can be employed [12, 13, 15]. Another possibility consists in optimizing the cDNA encoding the protein of interest according to the general codon usage of the host and the frequency of codons at 5′, which influence the translation efficiency [14, 16–18].

However, the expression of transport proteins is additionally hampered by their hydrophobicity, which strongly compromises expression throughput. Furthermore, eukaryotic membrane proteins are in average larger than bacterial counterparts [19] and are characterized by more complex hydrophilic moieties than the bacterial ones. Thus, bacterial folding machineries are inefficient for eukaryotic proteins [20], in particular for mammalian transporters, originated later in the evolution [21, 22]. It has been also reported that over expression in *E. coli* of eukaryotic membrane proteins results in saturation of the bacterial Sec translocon, leading to toxicity [20]. Exceptions to the higher complexity of eukaryotic proteins are the mitochondrial transporters which have been the first over expressed in bacteria [5, 23–25].

Other features of membrane transporters make them difficult to handle: water insolubility, higher mobility with respect to water-soluble proteins [26], and the propensity to form inclusion bodies as a consequence of inter- and intra-molecular aggregates in the bacteria cytosol. In other cases, the production of few molecules of heterologous proteins is toxic for the microorganisms, causing sudden cell death. This is the case of several amino acid transporters ([27], Galluccio and Indiveri unpublished results). *E. coli* C41, and C43 strains for toxic protein expression [27] or Lemo21 which should be effective in membrane protein expression [28, 29], have been developed from the original BL21.

In order to overcome problems linked to over expression of proteins, like membrane transporters, for which a general rule seems hard to be pointed out, a system that bypasses the choice of the appropriate combination host/plasmid has been established—the cell-free synthesis. Successful expression of membrane proteins by *E. coli* cell-free strategy was recently described [30, 31]. This system should give the advantages of reducing toxicity of expressed proteins and easier protein co-expression. However, in this case also, formation of insoluble aggregates was observed; thus, a modified strategy had been proposed based on protein translation into detergent micelles [32]. Eukaryotic in vitro translational systems from rabbit reticulocyte lysates [33] have also been pointed out. The main limit of cell-free expression systems, especially eukaryotic ones, is that even though they could give fast protein expression, the recovered amount is little, and normally not enough for structural studies. In few cases, bacterial cell-free expression led to appreciable results in terms of protein yield [30].

Another important challenge in protein over expression is the post-translational modification. Regarding this task, bacteria cannot perform, as example, the glycosylation of newborn proteins; however, several reports evidenced that glycosylation is normally not essential for transport activity but for other processes such as sorting in eukaryotic cells ([34] and refs herein [35]). Thus, the lack of this modification does not compromise functional and structural studies of transporters. Indeed, the few successfully over-expressed human (or mammalian) transporters in bacteria were functionally active [30, 36–39]. However, glycosylation could be obtained by over-expressing proteins in eukaryotic cells such as wheat germ extracts [33, 40], yeast [18] insects cell, such as Sf9 [33, 41]. Even though in these cases the glycosylation patterns are not always corresponding to those produced by human cells and the amount of recovered proteins is in general very low.

### Plasmid Selection

It is well known that promoter type and strength influence the levels of protein expression and that fusion tags may improve solubility of membrane proteins and purification. On the contrary, in the case of insoluble (membrane) proteins, high-strength promoter often leads to aggregation and inclusion body formation. Slowing down the expression rate in some cases allows membrane protein expression. Fusion tags are suitable for expression of hydrophobic proteins. Besides the most known 6, or longer, His tag, which is used essentially for purification [42], GST, MBP, NusA, Trx, and SUMO [43, 44] are potentially useful for improving solubility or even expression yield. For the expression of membrane proteins a promising approach might be the fusion with Mistic [45, 46], a small *E. coli* outer membrane protein, which can promote the insertion of the fusion peptide in the *E. coli* membrane to help the folding process. Chaperones co-expression has also been attempted to reduce formation of insoluble aggregates responsible for protein inactivation and difficult refolding. Several chaperones are commercially available, and used either alone or in combination, such as DnaKJE set or GroEL-GroES [47–49].

None of the above-described strategies revealed itself as a solution for general purposes of transport protein over expression. As a consequence, very few eukaryotic plasma membrane transporters have been heterologously over expressed so far [30, 36, 37, 39, 50, 51]. This has limited both the studies of the functional properties and the determination of the tertiary structures of membrane transport proteins which only recently started to be solved [52, 53].

One of the way to face transport protein expression may consist in dealing with experimental troubles with a group of proteins linked by common evolution, thus being structurally and functionally related, and optimize more than one critical factor among those described in the previous section. Indeed, related proteins will share similar responses to changes in protocols of expression and will have similar solubility properties and folding pathways, thus giving higher chances of success with at least one of the family member. Once optimal conditions are set up for one protein, it should be easier to carry out the expression of the others by minimal further changes of the experimental conditions. Based on this assumption we have focused our attention on the OCTN subfamily, which so far includes three proteins—OCTN1, OCTN2, and OCTN3, and a hypothetical splice variant OCTN2VT located in ER instead of plasma membrane [54]. A detailed overview of the strategies for obtaining these proteins in large amount, in a pure form, and appropriately folded will be provided, together with synthetic information on the functional and on the few structural information available on these proteins.

## Role of the OCTN Subfamily

The OCTN subfamily belongs to the SLC22 family, together with OCT and OAT subfamilies and, in the higher level of classification, to the Major Facilitator Superfamily to which more than thousand identified members belong, accounting for about 25 % of all transporter families [55].

The members of SLC22 family are characterized by broad substrate specificity: this started evolutionary studies to understand the origin of such variety. It has been proposed that the SLC22 family has evolved after the divergence of vertebrates and invertebrates [21]. The suggested mechanism is that of independent tandem duplications producing gene pairs. Since the duplicated gene products are functionally alike, gene pairing may be causative of the broader specificity observed in vertebrates compared to invertebrates. It has been speculated, furthermore, that the selective pressure on vertebrates coming from the more complex surrounding environment, led to a wider specificity toward several compounds [21].

## Function of the OCTN’s Proteins

**Fig. 1:**
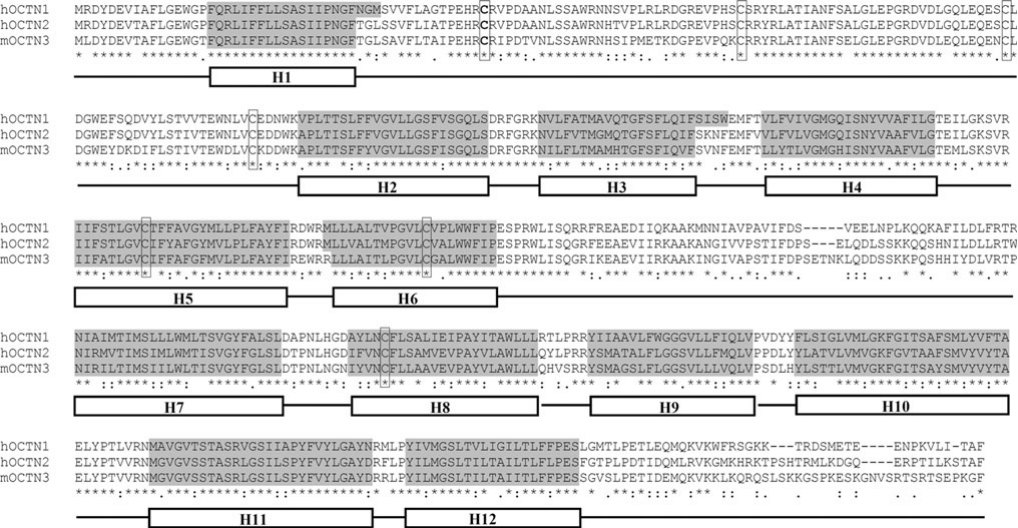
Alignment of proteins of the OCTN subfamily. Human OCTN1, human OCTN2, and mouse OCTN3 were aligned using the Clustal W software. Identities are indicated by *asterisks* and conservative or highly conservative substitutions are indicated by *dots* or *colons*, respectively. The Cys residues, which are conserved, are in *boxes*. Transmembrane segments predicted by TM-PRED software and manually adjusted for the three proteins, are indicated by *white boxes* and the corresponding sequences are highlighted in *gray*

Since the first identification of the cDNA coding for these proteins in human and mouse [56–60], it was shown that besides the structural relationships among the three proteins, a functional link also exists. Thus, the OCTN1-3 proteins are named organic cation transporters novel which are distinct from the OCTs [21, 59, 61]. The fact that at least some of the OCTN members recognize carnitine as substrate indicates that these transporters should be involved in the maintenance of the carnitine homeostasis in mammals [61–63]. This molecule does not have a precise classification since it was considered as vitamin up to the discovery of its biosynthesis pathway [64]. However, the biosynthesis does not produce sufficient amount for the whole body’s requirement; it may be then considered as a cofactor with, however, imprecise classification. Chemically, carnitine is a zwitterion essential for fatty acid β-oxidation in mitochondria and peroxisomes, and for VLDL assembly and protein modification in ER. Indeed, carnitine acts as shuttle molecule allowing the transfer of acyl units to CoA for the following oxidation reactions, while CoA, which cannot cross intracellular membranes, remains confined in the sub-cellular compartments. Moreover, carnitine is also involved in detoxification from drugs and xenobiotics since some of these compounds can form carnitine derivatives such as pivaloyl-carnitine, eliminated by kidney [62, 63, 65–69]. All these functions are essential for life, thus the intracellular carnitine concentration has to be strictly controlled and maintained constant. In mammals, the carnitine homeostasis is the result of endogenous synthesis, absorption from dietary source, renal re-absorption and excretion, distribution to tissues. This function is realized by a network of transporters and enzymes which is named carnitine system [63, 70] in which the three members of the OCTN subfamily very probably play important roles. In particular, it has been well established in several experimental systems that OCTN2 and OCTN3 accept carnitine as the main substrate while OCTN1 catalyses carnitine transport at a much lower efficiency; the OCTN1 and OCTN2 transport systems have a broad tissue expression demonstrated by RT-PCR analysis, Western Blot, and immunohistochemistry [56–63 and references herein]. The carnitine transport mediated by OCTN1 and OCTN2 has been reported to be dependent on Na^+^ gradient across plasma membrane, by analyzing the identified and cloned human and murine isoforms [56–60]. Before over expressing OCTN proteins [39, 50, 51], the functional and kinetic parameters have been evaluated, in several experimental models i.e., cell systems, intact brush border vesicles [56–60, 63, 71], as well as in reconstituted proteoliposomes [72]. The third member of OCTN subfamily, OCTN3 has been identified only in mouse and rat; thus, all the carried out studies concern the murine isoform, which shares high identity and similarity with the human OCTN1 and OCNT2 [71] (see Fig. 1; Table 1). In spite of the still lacking identification of the OCTN3 human gene, the OCTN3 protein has been detected in human tissues by western blot and immunohistochemistry using antibody raised against the murine isoform [71]. OCTN3 is a membrane transporter with a more narrow tissue expression profile compared to the other members; it is expressed, indeed, besides intestine, mainly in testis where it plays an important role in providing carnitine essential for spermatozoa development [73, 74]; in this district, in fact, its concentration is much higher than in plasma. The presence of OCTN3 has been shown also in brain where it may be involved in carnitine disposition in neurons. In these cells carnitine taken up by OCTN3 can help detoxification from drugs [75]. For OCTN1 and OCTN3 sub-cellular localizations, mitochondria and peroxisomes have also been proposed, respectively [76, 77]. Basing on studies conducted in cell systems and in intact vesicles, the carnitine transport mechanism described for mOCTN3 is pH and Na^+^-independent in a uniport mode [59].

**Table 1:** Comparison between the mouse and human members of the OCTN subfamily

	mOCTN1 (%)	mOCTN2 (%)	mOCTN3 (%)	hOCTN1 (%)
mOCTN2	75			
mOCTN3	71	84		
hOCTN1	85	73	68	
hOCTN2	72	85	78	76

Pairwise alignment have been performed by Clustal W. Percent identity are reported

The OCTN transporters, furthermore, have been proposed also to recognize and, sometime, transport other physiological compounds, such as organic cations, as well as some xenobiotics and drugs [56–61, 78, 79] in line with the broad substrate specificity mentioned above.

## Bacterial Over Expression of the Members of the OCTN Subfamily

**Fig. 2:**
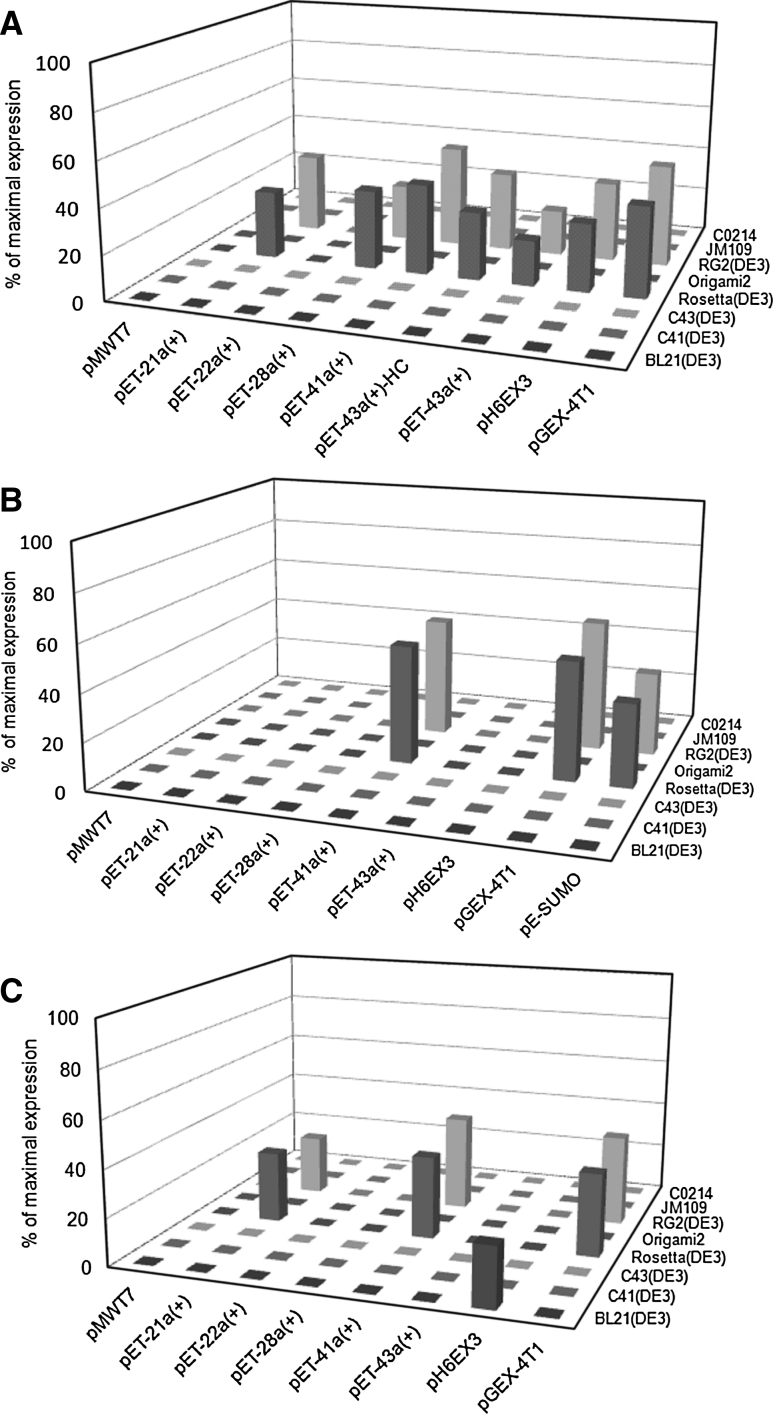
Screening of cell strains and plasmids for expression of OCTN proteins. Tridimensional representation of screening experiments for **a** hOCTN1, **b** hOCTN2, and **c** mOCTN3, in which different *E. coli* strains have been transfected with constructs obtained with each of the indicated plasmid. Strains are indicated by the commercial names except for RG2(DE3) which is the abbreviation of RosettaGami2(DE3). The histograms represent the expression level as percent of the maximal expression obtained for each of the protein [39, 50, 51] after optimization of all the experimental conditions

The first step of the study conducted on the OCTN subfamily proteins consisted in a screening strategy for checking the expression under different combinations of *E. coli* strains and plasmids constructed with the cDNA encoding each of the three proteins. As shown in Fig. 2, we succeeded in finding out some conditions in which each of the protein was expressed, even though at a low level, i.e., <50 % of the maximal expression obtained after optimization of the growth conditions [39, 50, 51].

### Strain–Plasmid Combination

The over-expression studies started from the first OCTN subfamily member, i.e., hOCTN1, then hOCTN2, and finally mOCTN3. From Fig. 2 it is evident that the most critical factor for expression was the choice of appropriate cell strains. In all cases only two of the tested strains gave some expression of the three proteins. The combination of pET-41a(+) or pGEX-4T1 with Rosetta(DE3) or Rosettagami2(DE3) led to the most significant expression of all the recombinant proteins. As reported in the scientific literature concerning membrane protein over expression, the presence of a fusion protein tag should increase the solubility of hydrophobic proteins. In the case of OCTN subfamily members we did not observe such effect; in fact the proteins were always present in the insoluble fractions of induced cell lysates. For hOCTN2, moreover, the plasmid pE-SUMO revealed to be suitable in over-expressing SUMOylated hOCTN2 (Fig. 2 and unpublished results). This result was probably due to a better recognition of the exogenous mRNA by the translation machinery of the bacteria.

The presence of the fusion tags had, however, the disadvantage of low recovery of protein after purification and separation procedure, even after optimization. In case of hOCTN1 a low but significant expression was also obtained with plasmids not carrying fusion proteins. pH6EX3 vector, indeed, revealed successful for obtaining a conspicuous expression of the recombinant protein. This protein construct contained the small 6His tag suitable for purifying the transporter. The best expression was obtained in RosettaGami2(DE3)pLysS, an engineered strain carrying two mutations in cytoplasmic disulfide reduction pathway to help disulfide bridges formation [50]. This gave the advantage to form appropriate disulfide(s) in the protein which revealed to be useful for refolding and functional reconstitution of the protein. As reported in Fig. 2, the screening on hOCTN2 and mOCTN3 has been conducted following the same criteria as for hOCTN1. Thus we have obtained an acceptable expression, around 30 % of the maximal, using pET-21a(+) in Rosetta(DE3) cell strain in case of mOCTN3. This plasmid carries a 6His tag at the C-ter of the recombinant protein which allowed the purification procedure on affinity column. Concerning hOCTN2 fused with GST, it was necessary to grow cells under osmotic stress conditions to obtain over expression of the interest protein. However, the highest yield was reached by applying codon optimization strategy. Based on the codon preferences of *E. coli*, the second triplet of hOCTN2 cDNA, CGG, was substituted with AAA, much more frequent in *E. coli* [14]. The AAA codon was chosen since it led to the conservative substitution R2K and, hence, very probably will not alter the structure/function relationship of the protein. The codon bias strategy has been significantly more successful in terms of protein yield compared to the one previously described. The modified cDNA was cloned in the pET-21a(+) and successfully expressed in Rosetta(DE3)pLysS, yielding hOCTN2R2 K with C-ter 6His tag, differently from hOCTN1 which had 6His tag at N-ter (not shown in Fig. 2).

### Growth and Inducing Conditions Optimization

After choosing the combination plasmid/host, several parameters influencing the expression were tested and optimized such as the inducer (IPTG) concentration, the post-induction temperature, and the growth time. It has been observed that slower growth, i.e., lower temperature or low IPTG concentration, led to higher expression, while faster growth caused lack of expression and/or cell growth arrest (toxicity) after a certain time. The best condition for hOCTN1, GST-hOCTN2, and mOCTN3 expression was obtained at 28 °C and 6 h of post-induction growth time [39, 50], while maximal amount of hOCTN2R2K was obtained after 4 h of induction [51]. The concentration of inducer (IPTG) was also optimized for the four constructs. For hOCTN1 and GST-hOCTN2, the best condition was 0.4 mM IPTG whereas mOCTN3 and hOCTN2R2K needed reducing the concentration of four folds, i.e., 0.1 mM IPTG for high-yield expression of protein. In all cases the proteins were present only in the insoluble fraction of induced cell lysate. Regarding the growth media used for the over expression among those tested, the LB medium was the best choice for hOCTN1, whereas 2X TY was used in case of hOCTN2R2K and mOCTN3. GST-hOCTN2 needed special growth conditions to recover high yield of recombinant protein; indeed, it was necessary to subject cells to an osmotic stress by adding sorbitol and betaine in the 2X TY medium. The induction of osmotic stress seems to cause metabolic changes in bacterial cells leading to better expression of the heterologous protein. In order to study the function and, if possible, the structure it is indispensable to obtain purified recombinant proteins. Then, the following step was the solubilization from pellet. In this case the three OCTN subfamily members showed very similar behaviors. Indeed a detergent screening has been performed in which several molecules were tested in the attempt to find out a non-ionic detergent which is able to solubilize hOCTN1, hOCTN2R2K, GST-hOCTN2, and mOCTN3 possibly in native forms, but without any significant result. However, these unsuccessful tries gave the opportunity to partially clean up the cell lysate from the soluble bacterial proteins which were removed by washing with non-ionic detergents. The mildest detergent which was able to solubilize the protein was the ionic sarkosyl. The minimal solubilizing sarkosyl concentration was 0.8 % [39, 50, 51].

### Purification Procedure and Protein Yield

Assuming that in the solubilized state the three proteins were unfolded, we searched for a procedure which could exchange the ionic for a non-ionic detergent. On the basis of previous experiences described by other groups [80], we set up a protocol for the purification of the protein which simultaneously exchanged the detergents. Thus, a Ni-chelating affinity chromatography was employed where the protein was tagged with 6His at the N-ter (hOCTN1and GST-hOCTN2) or C-ter (hOCTN2R2K and mOCTN3) [39, 50, 51]. After several attempts, a detergent/salts gradient was adopted in the chromatography, which led to recovery of protein soluble in Triton-X100. This indicated that some conformational rearrangements had occurred which most probably rely with the correct folding of the protein. Evidence for this hypothesis came later, when the hOCTN1 first and, then, mOCTN3 prepared with this procedure were successfully reconstituted in active form in proteoliposomes [38, 39]. The GST fusion in case of hOCTN2 has also been exploited with the aim of obtaining a less insoluble proteins which could be purified with an alternative method to Ni-chelating chromatography. Unfortunately, this was not the case of hOCNT2 because the purification yield was very low. Moreover, the GST-hOCTN2 fusion protein was again recovered in the insoluble fraction of cell lysate. The adopted purification strategies allowed recovery of the proteins with high yields. The highest amount was obtained for hOCTN2 after codon bias [51], which drastically increased the yield from a very low amount of 0.2 mg/l of cell culture with the GST-OCTN2 fusion protein to 3.5 mg/l of the hOCTN2R2K [51]. The low yield of purified hOCTN2 from GST-hOCTN2 was mainly due to the additional step of purification on size exclusion chromatography with Sephadex G-200 resin after thrombin cleavage of GST from hOCTN2. The yield of hOCTN1 was 3.0 mg/liter, while the yield of mOCTN3 was slightly lower than the other two members, i.e., 2.0 mg/l [39]. Taken together, all these data indicated that there are no straightforward criteria for improving the yield of even closely related proteins when expressing them in bacteria. Several critical and unidentifiable factors probably play roles in the interaction and processing of the mRNA/protein by the bacterial translation machinery. Indeed, in the case of hOCTN2 the simple change of the second codon revealed as *deus ex machina* to obtain over expression.

#### Apparent Molecular Mass of the Three Proteins and Relationships With the Structure (Sequence)

After purification, the three proteins were analyzed on SDS-PAGE. Immunodecoration using anti His-antibody was also employed to confirm the identification. Patterns on SDS-PAGE of the purified 6His-hOCTN1, hOCTN2-R2K-6His, and 6His-mOCTN3 showed that the apparent molecular masses of the three proteins were always about 10 kDa lower than the respective calculated masses [39, 50, 51] and slightly different among each other. This phenomenon has been already reported as a common feature of membrane proteins as “gel shifting” [80, 81]. These differences could not be easily explained on the basis of the physicochemical properties of the proteins especially in the light of the finding that single substitution in hydrophobic proteins causes strong variations of migration on SDS-PAGE [81]. Indeed, both the pI and the calculated molecular mass are not in line with the slightly different apparent molecular mass on SDS-PAGE of the three proteins (Table 2). mOCTN3 has an apparent smaller molecular mass with respect to OCTN1 and OCTN2 than expected from the very low difference of about 1 kDa in the theoretical mass. Thus, other factors should be involved in the migration shifts. Besides the high hydrophobicity of the proteins, it can be hypothesized that the observed general faster migration could be attributed to a compaction caused by formation of disulfides. The three OCTNs have, indeed, in their primary structure seven Cys residues which can form inter- and intramolecular disulfides. In previous reports ([81] and references herein) it has been shown that disulfides reduce SDS binding to globular proteins as well as membrane proteins and have been linked to a faster migration of oxidized forms compared to the reduced ones. In particular this has been demonstrated for hOCTN1. The treatment of the protein with 1 M β-mercaptoethanol, indeed, led to increase of the apparent molecular mass on SDS-PAGE up to the theoretical one [50]. The formation of disulfides in hOCTN1 may occur, mainly, among the three Cys residues located in three adjacent transmembrane segments, i.e., in a microenvironment less available for weaker reducing agent. Moreover, it is plausible that disulfide formation is important for the secondary structures maintenance which, on the other way round, hides the disulfide(s) itself. The smeared migration on SDS-PAGE of the over-expressed mOCTN3 could be still ascribed to different redox states of the protein [39]. Moreover, whether the redox status may or may not be related to the function of the proteins needs to be better investigated. On the other hand, the little variations of migration among the three proteins could be attributed to differences in the primary structure. A previously proposed hypothesis [81] is that more hydrophobic mutants generally bind more SDS leading to reduced gel mobility. This observation could be valid also for the three OCTN subfamily members since hOCTN1 shows a higher hydrophobicity with respect to the other two members (see Table 2). From the multi-alignment analysis depicted in Fig. 1 and from the data in Table 2, it can be observed that the percent in transmembrane segments is little different among the three proteins as well as the variations of charges in the non-conserved regions. These little differences might be determinant of the small mobility variations.

**Table 2:** Physicochemical properties of the members of the OCTN subfamily

	hOCTN1	hOCTN2	6His-mOCTN3	mOCTN3-6His
pI	7.11	7.66	8.32	8.51
Theoretical MW (kDa)	64.5	63.8	64.4	65.7
Apparent MW (kDa)	54	54	52	53
Hydrophobicity (%)	53	52	48	41

Data have been obtained with pI/pMW tool at the web site http://www.expasy.org

## Perspectives

### Functional Analysis and Relevance for Human Health

Notwithstanding the great number of publications dealing with OCTN subfamily members, their functions are still not completely understood. Even though OCTN’s are quite ubiquitously expressed and are described as plasma membrane proteins, in some reports different cellular and sub-cellular localizations have been shown. These discrepancies could also be explained by the existence of alternative splicing of the coding genes as previously suggested in evolutionary analysis [21]. In particular, for OCTN2 an alternative splice form has been described with different cellular localization and possible functions; it seems, in fact, that this isoform with an insertion of 24 amino acids does not transport carnitine [54].

The strategies described in this review revealed as a useful tool for over expressing the mammalian isoforms of OCTN subfamily members. The purified proteins, being soluble in the non-ionic detergent Triton X-100, could be used for both functional and structural studies upon recovery with high yield. This mild and non-denaturing condition results in a native form of the protein, suitable for further analysis. In this respect, it has to be highlighted that some functional studies have already been performed on purified hOCTN1 and mOCTN3 using liposomes to reconstitute the transport activity. The proteoliposome tool gives the advantage of studying the purified transporters in the absence of other transport proteins or enzymes otherwise present in cell systems. hOCTN1 was one of the first plasma membrane protein heterologously expressed to be reconstituted in liposomes [38]; the ability of the recombinant hOCTN1 to transport tetraethylammonium (TEA) and, with lower affinity, carnitine and ergothioneine has been shown. The transport was clearly inhibited by several organic cations and substrate analogs [38]. Noteworthy acetylcholine has been assessed to be a substrate of hOCTN1. The characteristics of the acetylcholine transport [82, 83] pointed out a new potential role of hOCTN1 in the non-neuronal cholinergic system, shedding new light on the potential pathophysiological role of OCTN1. Moreover, by this experimental approach some chemicals, reacting with Cys residues, have been evaluated for their inhibitory properties. It has been shown that almost all of them, in fact, are able to interact with the transporter, blocking substrate binding or transport reaction. This is in line with the presence of seven Cys residues in the primary structure of the transporter which might be able to interact with the compounds. These Cys are highly conserved (Fig. 1) among the OCTN subfamily members demonstrating their importance in maintaining the tertiary structure and the transport mechanisms. This is in line with the fact that Cys is one of the “gainer” amino acid, i.e., is one of the amino acids that increased along evolution of the protein [20]. hOCTN1 transport is regulated by physiological monovalent cations such as Na^+^ or K^+^. In the presence of extracellular Na^+^, hOCTN1 mainly catalyses acetylcholine efflux [83]. The reconstitution in liposome has also been used to study the recombinant mOCTN3 over expressed in *E. coli* [39]. Several data regarding the function of mOCTN3 previously studied in cell systems have been confirmed. Some new properties have been described such as, the inhibition by γ-butyrobetaine, precursor in the biosynthetic pathway of carnitine. This may play a role in regulating carnitine transport via mOCTN3. On a physiological point of view, mOCTN3 has been suggested to play a role in the carnitine network together with OCTN2 and, at lower extent, OCTN1 [63].

This system is essential for regulation of homeostasis particularly for those living organisms, like humans, in which more than half of the body’s carnitine is supplied by the diet [63, 68, 84, 85]. It is, then, not difficult to imagine that alterations of the carnitine homeostasis is responsible for a plethora of pathological states which range from severe syndromes caused by gene defects of transporters [2, 84] to light phenomena such as side effects of drugs which interfere with transporters [61, 63, 78, 86–88]. Among inherited alterations, some point mutations on OCTN2 coding gene have been clearly related to primary carnitine deficiency [OMIM 212140; 2, 86, 89, 90]; this is a recessively inherited disorder of fatty acid oxidation characterized by hypoketotic hypoglycemia, and skeletal and cardiac myopathy. The majority of natural mutations identified introduce a premature stop codon or impair insertion of the mutated transporter in plasma membrane; other missense mutations are responsible for a strong decrease in carnitine uptake due to change in transporter affinity toward the molecule or insensitivity to sodium stimulation, OCTN2 being a Na^+^-dependent transporter [86, 89, 90]. It has also been reported that some OCTN2 gene mutations are related to sudden infant death syndrome [91] with a specific cardiac and hepatic histopathology. OCTN1 is the less effective in carnitine transport among OCTN subfamily members and is involved in acetylcholine transport [82, 83]. However, genetic alterations of both OCTN2 and OCTN1 coding genes are associated with the onset of inflammatory bowel diseases (IBDs), such as Crohn’s disease [92], which are becoming more and more interesting in terms of development of possible treatments, since chronic inflammation may play a role in the pathogenesis of sporadic colorectal cancer. Moreover, it is known that OCTN1 polymorphisms may help to predict malignant progression of IBD [93, 94]. Acetylcholine has been shown to play a role in inflammatory processes [95]; thus transport of acetylcholine in a non nervous tissue, like intestine, is a new field of investigation. The third member of OCTN subfamily, even if OCTN3 has not yet been genetically identified in human genome, has been associated with male infertility in rat and in mice at different degrees of severity ranging from oligospermia to aspermia [75, 76, 96, 97], being mainly expressed in testis, besides intestine like OCTN1 and 2. Indeed, the carnitine concentration in epididymis is much higher (up to 2000 folds) [63] than in plasma suggesting that any alterations of carnitine transport in testis might be causative of homeostasis derangement. The carnitine homeostasis and its derangement is important also in cancer, since cancer cells show great metabolic changes, shifting from aerobic to anaerobic ATP production bypassing the mitochondrial function. In this scenario, carnitine has been suggested to play a regulatory function in the metabolic switch observed in cancer [63] and, thus, plasma carnitine transporters might be altered in their function or expression. Down regulation of OCTN2 in several epithelial cancers has been shown [98]. The fact that OCTNs are also expressed in brain where the transport mechanism of carnitine and its derivatives (with drugs as example) is not completely understood and definitively clarified is of great interest. Furthermore on a pathophysiological point of view, it is noteworthy that OCTN subfamily members have also been shown to interact and, sometimes, to transport [61, 78, 79, 87] a wide spectrum of xenobiotics, including drugs causing a reduction of carnitine or TEA transport. Some of these drugs may have inhibitory effect on substrate recognition and transport by the OCTN subfamily members, being a cause of slight alteration of carnitine metabolism in whole body, explaining some of the side effects of drugs; in some cases, in fact, those side effects mimic, in a milder form, the symptoms of primary carnitine deficiency [88–90]. These interactions in some cases may be due to disulfides formation between the compounds and Cys residues of the protein. In this respect, the generation of site-directed mutants of hOCTN1, hOCTN2, and mOCTN3 is important to understand the role of the single cysteine in transport mechanisms or regulations by chemicals.

The over expression in heterologous system followed by purification and reconstitution in liposomes can represent a good experimental tool for better understanding the functional properties of the natural mutants of OCTNs for which a clear association to human pathologies exists. Impaired acetylcholine efflux has been observed in the L503F mutant of hOCTN1 which is associated to Crohn’s disease [82]. The same strategy will allow functional and kinetic characterization of the OCTNs splicing variants; as suggested above some of these variants may have a different sub-cellular localization and/or function or may have tissue-specific distribution.

### Structural Analysis

**Fig. 3:**
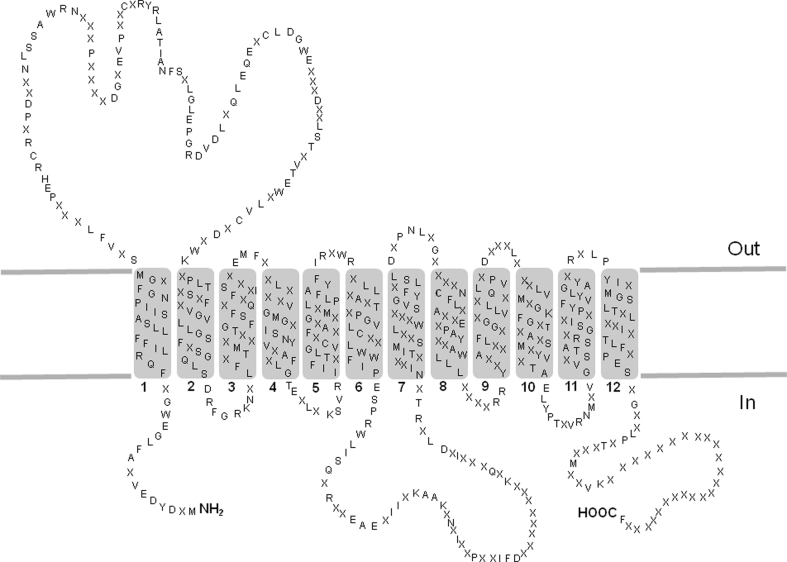
Topology of OCTN subfamily with 12 transmembrane α-helices and a large extracellular loop. The model was created on the basis of the TM-PRED prediction software (output format: min 17, max 29) and modified considering the established criteria for transmembrane proteins. Transmembrane segments are numbered (1–12). Residues which are identical in the three members (see also Fig. 1) are indicated by the respective *one letter* amino acid code; residues which are not conserved in all the proteins are indicated by *X*

The intrinsic flexibility together with the hydrophobicity of membrane transporters make them a great challenge for structural analysis. In fact despite the availability of advanced technologies for attempting such studies, there is currently a large imbalance between the number of solved tridimensional structures of soluble enzymes and that of membrane proteins; if we straiten the analysis to mammalian membrane transporters, the number of structures decreases dramatically. The structural studies need high amount of purified proteins and this, for membrane transporters, still represents a big limit. The purified protein recovered in high yield could be used for solving tridimensional studies. Hereto just bidimensional topological models have been proposed [63, 82]; a topology with 12 transmembrane helices has been predicted for the OCTN subfamily members (see Fig. 1). A big extracellular loop exists between the first and the second helix with potential glycosylation sites (three conserved Asn residues) and four Cys residues, which is supposed to be the most reactive with SH-reacting xenobiotics since it is the most exposed toward extracellular environment (Figs. 1, 3). The primary structure of this loop is quite well conserved among the three OCTNs. Moreover, according to these models, the N- and C-ter of the proteins as well a second loop, which is less conserved, between sixth and seventh transmembrane helix (Fig. 3) are intracellularly exposed [50, 63, 82]. The alignment in Fig. 1 shows that at this level hOCTN1 and hOCTN2 are more similar to each other than to mOCTN3. This could be explained by species-specific differences and by the fact that the second loop could be a potential site of intracellular regulatory pathways more or less active in humans or in mice. Some help in studying the transporter structure is given by homology modeling using, as template, known structures of other proteins. This has been previously done, as example, for rOCTs using the tertiary structure of Lac permease of *E. coli* as template [61]. Even though OCTs and OCTNs share some similarities, the tertiary structures of OCTN cannot be easily designed on Lac permease-deposited model essentially due to the difference in the large hydrophilic loop which is not present in Lac permease (http://www.rcsb.org/pdb). Among the secondary active transporter for which a structure in PDB bank has been recorded, there is the carnitine transporter CaiT from *E. coli* which catalyses exchange of L-carnitine with γ-butyrobetaine across *E. coli* membrane [99]. However, it was not possible to use this structure to model OCTNs, because the sequences of the transporters are too different. All together, this information shows the limits in solving tertiary structure of membrane transporters. It is plausible to believe that as for the over-expression strategies, attempting crystallization of proteins belonging to the same subfamily could also be helpful in finding successful methods.
